# Usability Evaluation of a Knowledge Graph–Based Dementia Care Intelligent Recommender System: Mixed Methods Study

**DOI:** 10.2196/45788

**Published:** 2023-09-26

**Authors:** Minmin Leng, Yue Sun, Ce Li, Shuyu Han, Zhiwen Wang

**Affiliations:** 1 Department of Nursing Shandong Provincial Hospital Affiliated to Shandong First Medical University Jinan China; 2 School of Nursing Peking University Beijing China; 3 Department of Cardiac Adult Postoperative Surgical Recovery Room, Fuwai Hospital National Center for Cardiovascular Diseases Chinese Academy of Medical Sciences Beijing China

**Keywords:** caregivers, dementia, knowledge graph, recommender system, usability evaluation, dementia care intelligent recommender system, DCIRS

## Abstract

**Background:**

Knowledge graph–based recommender systems offer the possibility of meeting the personalized needs of people with dementia and their caregivers. However, the usability of such a recommender system remains unknown.

**Objective:**

This study aimed to evaluate the usability of a knowledge graph–based dementia care intelligent recommender system (DCIRS).

**Methods:**

We used a convergent mixed methods design to conduct the usability evaluation, including the collection of quantitative and qualitative data. Participants were recruited through social media advertisements. After 2 weeks of DCIRS use, feedback was collected with the Computer System Usability Questionnaire and semistructured interviews. Descriptive statistics were used to describe sociodemographic characteristics and questionnaire scores. Qualitative data were analyzed systematically using inductive thematic analysis.

**Results:**

A total of 56 caregivers were recruited. Quantitative data suggested that the DCIRS was easy for caregivers to use, and the mean questionnaire score was 2.14. Qualitative data showed that caregivers generally believed that the content of the DCIRS was professional, easy to understand, and instructive, and could meet users’ personalized needs; they were willing to continue to use it. However, the DCIRS also had some shortcomings. Functions that enable interactions between professionals and caregivers and that provide caregiver support and resource recommendations might be added to improve the system’s usability.

**Conclusions:**

The recommender system provides a solution to meet the personalized needs of people with dementia and their caregivers and has the potential to substantially improve health outcomes. The next step will be to optimize and update the recommender system based on caregivers’ suggestions and evaluate the effect of the application.

## Introduction

According to Alzheimer’s Disease International [1], dementia affects more than 55 million people globally, and this number is expected to increase to 78 million by 2030 and 139 million by 2050. It is estimated that 15.07 million people aged 60 years or older in China live with dementia [2], accounting for approximately 25% of the global population with dementia [3]. The number of dementia cases in China is expected to reach 45.54 million by 2050 [4]. Due to the lack of effective treatment methods for dementia, personalized care for this expanding patient population to slow the progression of the disease is critical. In long-term health maintenance, caregivers are faced with complicated and diversified care problems such as managing activities of daily living [5,6], behavioral and psychological symptoms [7,8], safety risks [9,10], and attending to their own mental health [11,12]. However, due to a lack of supportive resources and knowledge about dementia, caregivers have low confidence in managing caregiving and do not know what to do when people with dementia exhibit abnormal behavior [13-15]. In addition, inappropriate care behavior can accelerate the disease deterioration of care recipients. As a result, caregivers of people with dementia experience higher levels of ineffective coping, psychological burden, and physical strain [16,17]. Given the serious impact on the physical and mental health of caregivers, effective and practical support for caregivers is essential.

The rapid development of internet-based interventions has promoted the inclusivity and universality of dementia care support services. Internet-based interventions are relatively low cost and more accessible to caregivers, especially those who live in remote areas or find it difficult to leave their care recipients, and they increase equal access health care [18,19]. Such internet-based interventions have gradually been applied to people with dementia [20,21] and their caregivers [22-24], but most of these interventions are more general in nature, offering universal support services. Studies have shown that personalized internet-based supportive interventions are more effective and favored by caregivers [25,26]. Currently, there are personalized internet-based support interventions for caregivers of people with dementia, such as “Caregivers’ Friend: Dealing with Dementia,” developed by Beauchamp et al [27] and “A Technology Platform for the Assisted Living of Dementia Elderly Individuals and Their Carers,” developed by Torkamani et al [28]. Caregivers can interact with dementia care experts in real time by clicking the “Guide Me” or “Contact Me” button to obtain personalized guidance. In the “Care Ecosystem” intervention developed by Possin et al [29], care team navigators respond to caregivers’ immediate needs first, and then screen for common problems and provide personalized support and standardized education. In the “FamTechCare” intervention developed by Williams et al [30], caregivers are provided with a telehealth video-monitoring unit (iPad Mini with the behavior capture app). The app uses a buffering technology to capture antecedents leading to a challenging care situation. Caregivers upload videos for review by an expert team. The dementia care experts address the care dyad by providing tailored feedback based on specific care encounters. The above personalized internet-based guidance requires real-time web-based support from dementia care experts or matching monitoring equipment.

However, due to the insufficient number of multidisciplinary experts in the field of dementia in China, it is difficult to provide one-to-one real-time guidance to a large number of dementia caregivers, so the personalized and diversified needs of caregivers are not met. In this case, a knowledge graph–based recommender system can provide a solution [31,32]. The knowledge graph represents various entities and their relationships in the domain with the “entity-relationship-entity” triplet, and the entities are connected by their relationships to form a network knowledge structure [33], which is gradually used in the disease knowledge question-answering system [34]. Knowledge graphs can provide efficient data representation for recommender systems, overcome the problems that exist in traditional recommender systems, such as sparse data, cold startup, and a lack of data semantic information mining, and improve the accuracy, diversity, and interpretability of recommendation results [32,35]. In recent years, knowledge graph–based recommender systems have developed rapidly and have been widely applied for recommending movies, music, news, commodities, etc [36-38]. In the past 2 years, they have also been preliminarily applied for target drug recommendations [39], disease diagnosis [40], and treatment planning [41]. To provide personalized support services for caregivers of people with dementia, our research team developed a knowledge graph–based dementia care intelligent recommender system (DCIRS). The DCIRS can push personalized care plans for caregivers according to the unique characteristics and care problems of a patient with dementia.

Usability evaluation is an indispensable link in the process of electronic product development, one of the key factors in the successful implementation of telemedicine, and a critical means of driving adoption and improving user compliance [42,43]. According to the International Organization for Standardization 9241-11, usability is defined as the effectiveness, efficiency, and user subjective satisfaction with a system, product, or service when it is used for specific goals by specific users in a specific context of use [44]. These metrics can be measured by gaining insight into user perceptions of performance, acceptability, and satisfaction when using the system, product, or service [45]. Evaluating usability is of tremendous value to developers and users, so it is recommended by some scholars that a certain amount of time and resources should be invested in usability evaluation before conducting large clinical trials [46-48]. Therefore, this study aimed to evaluate the usability of the DCIRS with the goal of identifying the potential user interface, functionality, ease of use, and user willingness to engage from the perspective of caregivers.

## Methods

### Overview

This study followed the IDEAS (Integrate, Design, Assess, and Share) framework for developing digital health behavior change interventions [42]. According to the IDEAS framework, rigorous large-scale randomized controlled trial evaluations of digital products should be preceded by small-scale evaluations to test potential efficacy and usability. Specifically, a questionnaire can be used to evaluate usability and satisfaction, and interviews can be conducted to understand user experiences.

### Study Design

A convergent mixed methods design was used to collect usability data. A standardized quantitative survey and semistructured interviews were administered to obtain a more comprehensive understanding of the perceived usability of the DCIRS among dementia caregivers. Compared with other mixed methods designs, such as explanatory mixed methods design, exploratory mixed methods design, and embedded mixed methods design, convergent mixed methods design can compensate for the weaknesses of 1 type of data with the advantages of another. The 2 types of data complement each other, which not only expands the research breadth but also increases the research depth [49].

### Participants

From January 12 to January 18, 2022, participants were recruited to use the DCIRS through advertisements displayed on a WeChat official account named “Care for Dementia with You.” Before the study began, all participants were informed of the purpose of the study and signed an informed consent form. Participants were eligible to participate if they met the following inclusion criteria: (1) were caregivers who were currently providing care to people living with dementia and would continue to care for the person for at least four weeks; (2) had care recipients with a definite diagnosis of dementia and the disease stage (dementia was clinically diagnosed according to the Diagnostic and Statistical Manual of Mental Disorders, Fourth Edition [DSM-IV] criteria and the severity of dementia [mild, moderate, and severe dementia] was measured using the Clinical Dementia Rating scale); (3) had an education level of primary school or more and had usual access to smartphones, tablets, or laptops; (4) could provide detailed personal and disease-related information about the care recipients; (5) were ≥18 years old; and (6) volunteered to participate in this study and give feedback about their experience. For the sample size, Bastien [50] cited studies showing that most usability problems can be found in a sample of 5-15 participants. As Virzi [51] showed, only 4-5 participants are needed to identify about 80% of usability problems, and this number is sufficient to reveal the most severe problems. Assuming a dropout rate of 20% for the clinical sample, at least 18 participants needed to be invited to participate in the study. However, considering the saturation of qualitative interview information and the maximum diversity of sample selection, for the sake of being conservative, we expanded the sample by 3 times, that is, at least 54 participants were invited to enroll in the study.

### Development of DCIRS

#### Overview

In the first stage, our research group worked with computer engineers to construct a knowledge graph of dementia care. Based on the 1012 real cases of dementia investigated by the research group, the multidisciplinary team formulated personalized care plans as the dementia care case base. The dementia care case base was based on real clinical cases and collated evidence from standardized clinical guidelines and systematic reviews, as well as the practical experience of experienced caregivers and multidisciplinary experts. Then, using the dementia care case base as the knowledge source, knowledge extraction technology was used to extract entities and interentity relationships to obtain “entity-relationship-entity” triplet data, which were ultimately stored in the Neo4j graph database to complete the construction of the knowledge graph. The constructed knowledge graph of dementia care takes people with dementia as the core and unfolds, one by one, around personalized characteristics, daily living care problems, behavioral and psychological symptoms, safety risks, the arrangement of the living environment, the arrangement of activities, and the corresponding care advice for specific care problems in a standardized “entity-relationship-entity” triplet format, forming a large knowledge network. Due to the large capacity of the constructed knowledge graph, part of the visualization is presented in Multimedia Appendix 1.

In the second stage, the established knowledge graph of dementia care was introduced into the recommendation model by way of graph embedding to form a recommendation model composed of a graph embedding module and a recommendation module. The graph embedding module learns the features of the knowledge graph, and the recommendation module interacts with the features of the knowledge graph (learned in the graph embedding module) and items in the recommendation module through intelligent algorithms to yield personalized recommendations for the care plan.

#### User Side

The core function of the DCIRS is to recommend a personalized care plan according to information about the individual user and consists of 2 modules: the “comprehensive evaluation” and “personalized care plan query” modules. The DCIRS also adds 3 auxiliary modules: the “personalized question-answering,” “typical cases,” and “common questions and answers” modules. The function introduction of each module and the core function display are as follows:

1. Comprehensive evaluation: this module mainly evaluates the personalized characteristics of people with dementia (eg, sex, severity of dementia, hobbies, and walking ability) and care problems in 3 aspects: daily living care problems, behavioral and psychological symptoms, and safety risks. Caregivers complete the assessment based on the condition of the care recipient. The operation flow interface of the “comprehensive evaluation” module is shown in Multimedia Appendix 2.
2. Personalized care plan query: after completing the assessment in the “comprehensive evaluation” module, caregivers can query the personalized care plan that has been reviewed and approved by 1 or 2 dementia care experts within 24 hours after submitting the assessment. The personalized care plan mainly involves the arrangement of the living environment, the arrangement of activities, and the corresponding care advice for specific care problems. The operation flow interface of the “personalized care plan query” module is shown in Multimedia Appendix 3.
3. Personalized question-answering: with the help of natural language processing technology, the caregiver can query the coping method by entering keywords regarding care problems.
4. Typical cases: the research group summarized some typical cases and their coping methods based on actual cases, including avoiding excessive care, diet care, sleep promotion, abnormal behavior responses, the caregivers’ own psychological adjustment, and COVID-19 prevention, for caregivers to browse and learn according to their own interests.
5. Common questions and answers: based on our previous research [52-54], the research team summarized some issues that caregivers are concerned about, such as prevention of dementia, early identification of dementia, and coping methods for some specific problems that commonly occur in people living with dementia, for caregivers to browse and learn according to their own interests.

#### Backstage Management

The backstage management of DCIRS includes the management of data, knowledge graph, typical cases, common questions and answers, questionnaires, return visits, and administrators. Data management is mainly for personalized care plan management. Through the personalized information entered by the user, the DCIRS will automatically push the personalized care plan suitable for individuals with dementia in the backstage. After the plan is reviewed and approved by the dementia care expert team, the user can submit queries about the plan by clicking on an icon in the DCIRS. Knowledge graph management can update knowledge by adding new entities, interentity relationships, and new cases of dementia to provide support for “personalized question-answering” on the user side. Typical case management and common question-and-answer management can add or modify new case or question problems to continuously enrich and update the knowledge about dementia care. Questionnaire management and return visit management are mainly used to manage the data collected based on user feedback in the later stage. Administrator management is mainly used to manage dementia care experts and programmers. The interface for backstage management is shown in Multimedia Appendix 4.

### Procedures

A backstage management team consisting of 2 research assistants, 2 dementia care experts, and 1 software engineer was established to ensure the normal operation of the DCIRS. The research assistants were responsible for user management, statistics, and the backup of backstage data, involving the following aspects: recruiting participants; monitoring and managing the normal use of all login users; and organizing, counting, and backing up backstage data. The dementia care experts were responsible for the review and release of personalized care plans; after a user completed the assessment in the “comprehensive assessment” module, the dementia care experts reviewed whether the personalized care plan automatically generated by the DCIRS was accurate based on the comprehensive assessment of people with dementia and their own professional knowledge reserve. The precise personalized care plan was then pushed directly to the user. If there was inappropriate care advice in the care plan, it was modified and then pushed to users. The dementia care experts completed the review, revision, and release of the personalized care plans within 24 hours, so that users could query personalized care plans in the “personalized care plan inquiry” module in a timely manner. The software engineer ensured the normal operation of the DCIRS, which involved the following aspects: monitoring whether there were code program–related problems in the backstage data, solving problems in a timely and efficient manner to ensure the normal operation of the DCIRS, and performing daily maintenance of the DCIRS. The recruited participants were first informed of the purpose of the study, and then the website link to the DCIRS was sent to them, and the method of using the DCIRS was introduced. Subsequently, participants used the DCIRS for 2 weeks. Participants could also reach the research assistants by phone or WeChat if they encountered any problems using the DCIRS. After 2 weeks of use, the research assistants evaluated the usability of the DCIRS with a scale and semistructured interviews.

### Data Collection

#### Quantitative Data

The Computer System Usability Questionnaire (CSUQ) was used to assess usability among all DCIRS users [55]. The CSUQ is a reliable and valid usability scale and provides a global view of the subjective usability of a website, software, system, or product at the end of a study. It is a 19-item standardized tool containing 4 components, including system usefulness (items 1-8), information quality (items 9-15), interface quality (items 16-18), and overall satisfaction (items 1-19). Each item is scored on a 7-point Likert scale, ranging from 1 (strongly agree) to 7 (strongly disagree). The overall result is calculated by averaging the scores of all items, and lower scores indicate higher usability. The sociodemographic characteristics of caregivers (age, sex, education level, years of care experience, average care hours per day, and relationship with care recipients) and care recipients (age, severity of dementia, and disease duration) were also collected to describe the sample.

#### Qualitative Data

Semistructured interviews were conducted to collect additional feedback and comments on users’ experiences to gain more detailed insight into the usability of the DCIRS. Caregivers were included until there was a maximum variation in the participant characteristics (age, sex, education level, years of care experience, setting, relationship with care recipients, and dementia severity of care recipients) to make the collected feedback more representative. Interviews were conducted by a researcher who was a PhD student and trained in qualitative research techniques. Each interview took approximately 30 minutes. The number of interviewees was judged by information saturation [56]. When the researcher found that no new feedback information could be obtained from the 2 interviewees, the interview information was considered to be saturated. All interviews were audio-recorded and later transcribed by the researcher to identify common themes. The specific interview guide is as follows: (1) What was your overall feeling when you used this recommender system? (2) What do you think the advantages of this recommender system are? What beneficial experience has it provided to you? (3) What do you think the disadvantages of this recommender system are? How would you propose to improve or optimize this system? (4) and are you willing to continue to use this recommender system to assist you in caring for people living with dementia? Why?

### Data Analysis

#### Quantitative Data

Descriptive statistics were used to describe sociodemographic characteristics and CSUQ scores. Categorical variables, including sociodemographic characteristics, were described as frequencies and percentages. Continuous variables, including sociodemographic characteristics and CSUQ scores, were described as the means and SD. Quantitative data were analyzed using IBM SPSS Statistics version 22.0 (IBM Corp).

#### Qualitative Data

After each interview, the researcher who conducted the interview listened to the audio recording within 24 hours, transcribed it, and imported it into qualitative analysis software NVivo 12.0 (QSR International). The transcripts were analyzed systematically using inductive thematic analysis as described by Braun and Clarke [57]. First, 2 researchers read the transcripts repeatedly to become familiar with the overall data. Second, open coding was performed by 2 researchers independently to develop the initial codes for the data. Next, all transcripts were reread and cross-checked for the assigned codes, and any disagreements were resolved by a third researcher. Afterward, codes with similar content were merged and grouped to form subthemes. Subsequently, the subthemes were clearly defined and categorized within the main themes, which reflected what they represented. After these steps, selected quotes were provided as examples of identified subthemes, which were discussed by the researchers.

#### Mixed Methods Integration and Analysis

The data were integrated with the following steps: quantitative analysis; qualitative analysis; identification of similar and dissimilar results; and confirmation, expansion, or discordance of the results [49]. Confirmation occurred if the findings from both types of data reinforced the results from the other. Expansion occurred when the findings from the 2 data sets diverged and expanded insights into usability by addressing different or complementary aspects of the user experience. Discordance occurred if the survey and interview results were inconsistent, contradictory, or disagreed with each other [58].

### Ethics Approval

This study was approved by the Biomedical Ethics Committee of Peking University (ethics approval number: IRB00001052-21095). Before the study began, all participants signed an informed consent form. Participants were told that their participation in the study was voluntary, that they could withdraw or stop at any time, and that all data would be kept strictly confidential and only the researchers would have access to it.

## Results

### Quantitative Results

A total of 56 caregivers who met the inclusion criteria were recruited, and all completed the use of the DCIRS. In the data collection stage, after 2 weeks of use, 53 questionnaires were collected, for a recovery rate of 94.64%.

#### Sociodemographic Characteristics of the Participants

Most of the caregivers were female (n=41, 77%), and most were the daughters or sons of the care recipients (n=35, 66%). The mean ages of the caregivers and care recipients were 48.40 (SD 10.95) years old and 74.75 (SD 7.79) years old, respectively. The detailed characteristics of the caregivers and their care recipients are shown in Table 1.

**Table 1:** Sociodemographic characteristics of caregivers and care recipients (n=53).

Sample characteristics		Values
**Sex, n (%)**		
	Male	12 (23)
	Female	41 (77)
Age (years), mean ( SD)		48.40 (10.95)
Age of care recipients (years), mean ( SD)		74.75 (7.79)
Disease duration of care recipients (years), mean ( SD)		4.62 (2.63)
Average care hours per day (hours), mean ( SD)		13.74 (8.71)
Care experience (years), mean ( SD)		4.22 (2.44)
**Care recipients’ dementia severity, n (%)**		
	Mild	10 (19)
	Moderate	32 (60)
	Severe	11 (21)
**Education level, n (%)**		
	Primary school	3 (6)
	High school	12 (23)
	University	28 (53)
	Master’s degree or above	10 (19)
**Relationship with care recipient, n (%)**		
	Daughter or son	35 (66)
	Spouse	9 (17)
	Daughter-in-law or son-in-law	3 (6)
	Other	6 (11)

#### CSUQ Scores

The mean CSUQ score (2.14) suggested that the DCIRS was generally easy for caregivers to use. Among the 19 items of the CSUQ, the top 3 items with positive evaluations were item 8, “I believe I became productive quickly using this system” (mean 1.60, SD 0.91); item 13, “The information provided with the system is easy to understand” (mean 1.64, SD 0.90); and item 7, “It was easy to learn to use this system” (mean 1.77, SD 0.87). The top 3 negative evaluation items were item 18, “This system has all the functions and capabilities I expect it to have” (mean 2.75, SD 1.07); item 9, “The system gives error messages that clearly tell me how to fix problems” (mean 2.57, SD 1.05); and item 5, “I am able to efficiently complete my work using this system” (mean 2.43, SD 1.01). The 4 dimensions of the CSUQ scored in order from best to worst were system usefulness, information quality, overall satisfaction, and interface quality. The specific scores for each item and the 4 components are shown in Table 2.

**Table 2:** Computer System Usability Questionnaire scores of each item (n=53).

Items			Score, mean (SD)
**System usefulness (items 1-8)**			2.05 (0.80)
	1. Overall, I am satisfied with how easy it is to use this system	1.79 (0.91)	
	2. It is simple to use this system	1.91 (0.88)	
	3. I can effectively complete my work using this system	2.32 (1.02)	
	4. I am able to complete my work quickly using this system	2.40 (0.95)	
	5. I am able to efficiently complete my work using this system	2.43 (1.01)	
	6. I feel comfortable using this system	2.17 (0.87)	
	7. It was easy to learn to use this system	1.77 (0.87)	
	8. I believe I became productive quickly using this system	1.60 (0.91)	
**Information quality (items 9-15)**			2.11 (0.83)
	9. The system gives error messages that clearly tell me how to fix problems	2.57 (1.05)	
	10. Whenever I make a mistake using the system, I recover easily and quickly	2.30 (1.03)	
	11. The information (such as on-line help, on-screen messages, and other documentation) provided with this system is clear	1.91 (0.88)	
	12. It is easy to find the information I need	2.09 (0.99)	
	13. The information provided with the system is easy to understand	1.64 (0.90)	
	14. The information is effective in helping me complete my work	2.04 (1.00)	
	15. The organization of information on the system screens is clear	2.25 (0.81)	
**Interface quality (items 16-18)**			2.47 (0.86)
	16. The interface of this system is pleasant	2.26 (0.92)	
	17. I like using the interface of this system	2.40 (0.97)	
	18. This system has all the functions and capabilities I expect it to have	2.75 (1.07)	
	19. Overall, I am satisfied with this system	2.04 (0.98)	
Overall satisfaction (items 1-19)			2.14 (0.79)

### Qualitative Results

Data saturation was reached after 16 consecutive interviews with participants. Detailed characteristics of the caregivers and care recipients are shown in Table 3. The thematic analysis found 14 subthemes, which were grouped into 4 themes: the overall experience using the DCIRS, the advantages and beneficial experience of the DCIRS, the shortcomings of the DCIRS and optimization suggestions, and the willingness to continue using the DCIRS. The analysis of the interview content is provided in Table S1 in Multimedia Appendix 5.

**Table 3:** Sociodemographic characteristics of caregivers and care recipients (n=16).

Sample characteristics		Values
**Sex, n (%)**		
	Male	3 (19)
	Female	13 (81)
Age (years), mean ( SD)		49.44 (13.78)
Age of care recipients (years), mean ( SD)		77.69 (6.38)
Disease duration of care recipients (years), mean ( SD)		3.69 (2.21)
Average care hours per day (hours), mean ( SD)		13.06 (9.06)
Care experience (years), mean ( SD)		3.44 (2.02)
**Care recipients’ dementia severity, n (%)**		
	Mild	4 (25)
	Moderate	8 (50)
	Severe	4 (25)
**Education level, n (%)**		
	Primary school	1 (6)
	High school	2 (12)
	University	10 (63)
	Master’s degree or above	3 (19)
**Relationship with care recipient, n (%)**		
	Daughter or son	9 (56)
	Spouse	3 (19)
	Daughter-in-law or son-in-law	1 (6)
	Other	3 (19)

### Mixed Methods Results

The themes that emerged from the qualitative results were compared and merged with the quantitative results in terms of the overall experience using the DCIRS, the advantages and beneficial experience of the DCIRS, the shortcomings of the DCIRS and optimization suggestions, and the willingness to continue using the DCIRS. Quantitative data showed that the overall usability of DCIRS was relatively high, especially scores on item 8 (“I believe I became productive quickly using this system”), item 13 (“The information provided with the system is easy to understand”), and item 7 (“It was easy to learn to use this system”), but the interface quality score was somewhat poor. On this basis, specific usability issues (advantages, disadvantages, and optimization suggestions) of the DCIRS were deeply explored through qualitative interviews. The advantages included the content of the DCIRS, which was professional, easy to understand, instructive, met their personalized needs, etc. The disadvantages and optimization suggestions included poor aesthetics and a need for more functions such as professional-caregiver interactions, caregiver support, and resource recommendations. Quantitative results and qualitative results were generally consistent, and no discordant results were observed. The comparison and merging of the 2 data sets resulted in confirmed and expanded findings.

## Discussion

### Principal Findings

In this study, a mixed methods approach was used to conduct a usability evaluation of the DCIRS through a CSUQ survey and semistructured interviews. Quantitative data suggested that the DCIRS is easy for caregivers to use, with a mean CSUQ score of 2.14. Qualitative data showed that caregivers generally believed that the content of the DCIRS was professional, easy to understand, instructive, and could meet their personalized needs; they were willing to continue to use it. However, it also had some shortcomings, such as poor aesthetics and a need for more functions such as professional-caregiver interactions, caregiver support, and resource recommendations.

The knowledge graph–based DCIRS developed by our research team can push personalized care plan recommendations for caregivers according to the characteristics and care problems of individual patients with dementia. A knowledge graph can provide efficient data representation, facilitate the recommender system to mine user preferences, and improve the accuracy, diversity, and interpretability of recommendations [32,38]. The characteristics and care problems of people with dementia are complex and diverse, so the care plan also varies according to the person. The knowledge graph–based recommender system provides the opportunity to realize personalized dementia care plans. The knowledge of the DCIRS mostly comes from standardized clinical guidelines [59,60], systematic reviews [61,62], practical experience of experienced caregivers and multidisciplinary experts, and real clinical cases, and it prevents problems such as unprofessional information and poor accuracy. The new data and knowledge generated in the future can be quickly integrated into the existing knowledge graph to realize the continuous updating and accumulation of dementia care knowledge.

Although the majority of caregivers expressed satisfaction with the overall design and user-friendliness of the DCIRS, a number of areas for improvement were identified through user feedback and will be implemented in future designs. To improve the usability of the DCIRS, the main changes that need to be implemented include enhancing the aesthetics, adding functions for professional-caregiver interactions, caregiver support, and resource recommendations, and classifying the knowledge of auxiliary modules according to disease stages or caregivers’ experience level. In addition, the DCIRS will be optimized and upgraded according to the usability evaluation feedback. The users will be dynamically evaluated and continuously tracked in the formal use stage, and the health improvement of people with dementia will be measured by comparing the results before and after the evaluation. Usability evaluation plays an irreplaceable role in the development of web-based platforms. Many web-based platforms, such as software [63,64], websites [65,66], systems [67,68], and other products [48,69], have been evaluated for usability before large-scale clinical trials are conducted. The findings of the usability evaluation in this study can provide reference and guidance for the development of similar web-based platforms in the field of dementia care and raise other developers’ awareness of possible shortcomings in their own web-based platforms.

### Implications and Future Work

The research team will optimize and upgrade the DCIRS according to the caregivers’ feedback and conduct a large sample clinical trial to explore the effectiveness and cost-effectiveness of the intervention program. In terms of the optimization of intelligent algorithms, users will be dynamically evaluated, and feedback will be collected regularly during the use period to evaluate whether the pushed care plan is effective and which specific care advice is effective. On the basis of considering the individual characteristics of people with dementia, care advice that is useful according to users’ feedback will be pushed first, while care advice that users deem less useful will follow. The accuracy of the recommendations will be improved with the help of intelligent algorithms combined with feedback data. In addition, the influence of the implementation of personalized care plans on the effect will be further explored to promote intelligent care and practice.

### Limitations

This study has several limitations. First, for the usability evaluation, a standard formula for calculating the sample size was not found. The sample size of this study was roughly estimated by referring to previous similar studies. Second, ensuring that the recommended care plan is effectively implemented is challenging. Finally, this study only conducted a usability evaluation and not an application effect evaluation. In the next step, a rigorous randomized controlled trial should be designed to evaluate the effect of the DCIRS.

### Conclusions

This study demonstrated that the prototype of the DCIRS is available, feasible, and easy to use for caregivers of people with dementia. The DCIRS provides a solution to meet the personalized needs of people with dementia and their caregivers and has the potential to substantially improve health outcomes. The next step will be to optimize and upgrade the DCIRS based on the caregivers’ suggestions and promote the use of the DCIRS as a means of providing intelligent care in the field of dementia care.

## Appendix

Multimedia Appendix 1 Knowledge graph of dementia care (part).

Multimedia Appendix 2 The operation flow interface of the "Comprehensive evaluation" module.

Multimedia Appendix 3 The operation flow interface of the "Personalized care plan query" module.

Multimedia Appendix 4 The interface of backstage management.

Multimedia Appendix 5 Analysis of the interview content.

## Abbreviations

CSUQ: Computer System Usability Questionnaire
DCIRS: dementia care intelligent recommender system
DSM-IV: Diagnostic and Statistical Manual of Mental Disorders, Fourth Edition
IDEAS: Integrate, Design, Assess, and Share
