# Supplementary figures and images for: Usability Evaluation of a Knowledge Graph–Based Dementia Care Intelligent Recommender System: Mixed Methods Study
Source: J Med Internet Res. 2023 Sep 26;25:e45788. doi: 10.2196/45788 (PMC10565620; doi:10.2196/45788)

**Multimedia Appendix 1**


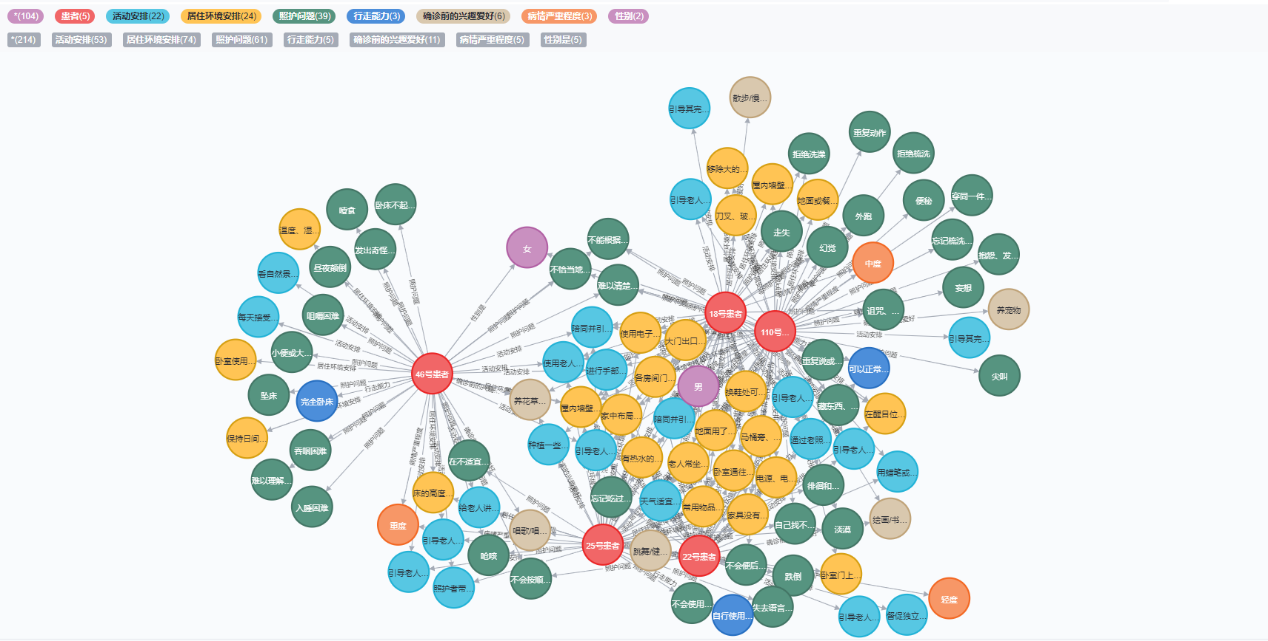


**Figure S1.** Knowledge graph of dementia care (part).

Supplement: Multimedia Appendix 1 [file jmir_v25i1e45788_app1.docx]
