# Supplementary material for: Usability Evaluation of a Knowledge Graph–Based Dementia Care Intelligent Recommender System: Mixed Methods Study
Source: J Med Internet Res. 2023 Sep 26;25:e45788. doi: 10.2196/45788 (PMC10565620; doi:10.2196/45788)
Supplement: Multimedia Appendix 2 [file jmir_v25i1e45788_app2.docx]

**Multimedia Appendix 2**


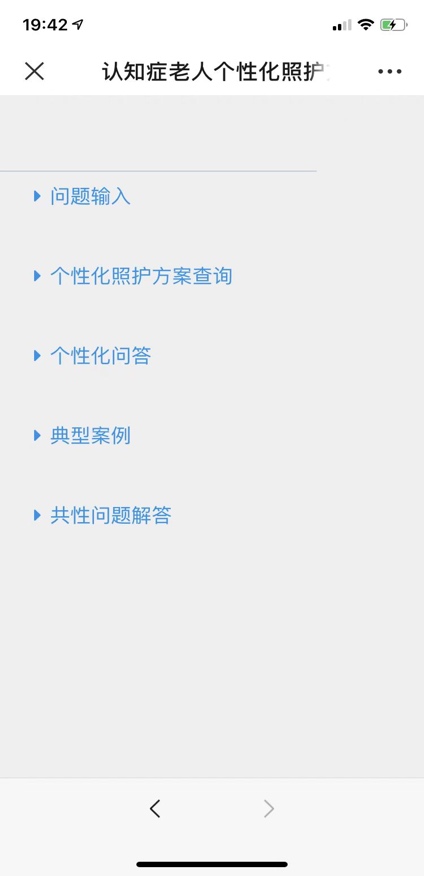


**Dementia care intelligent recommender system**

**Comprehensive evaluation**

**Personalized care plan query**

**Personalized question-answering**

**Typical cases**

**Common questions and answers**

**Information input**


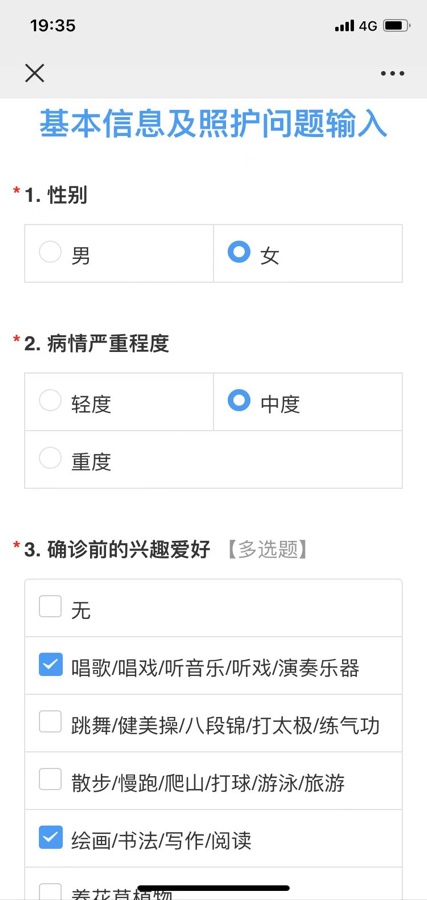


**1. Sex**

Female

Male

**2. Severity of dementia**

Mild

Moderate

Severe

**3. Hobbies before the diagnosis of dementia**

None

Dancing/dancing aerobics/dancing baduanjin /doing Tai Chi/doing Qigong

Singing/listening to music/playing musical instruments

Take a walk/running/climbing/playing ball/swimming/traveling

Painting/calligraphy/writing/reading


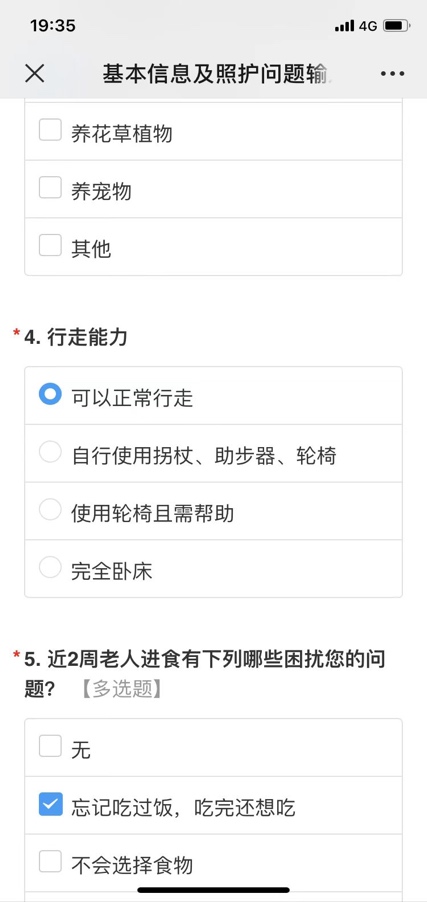


Growing flowers and grass

Keeping pets

Other

**4. Walking ability**

Can walk normally

Self-use of crutches, walkers, wheelchairs

Using a wheelchair and requiring assistance

**5. In the past two weeks, which of the following problems troubled you about the diet of dementia people?**

Be completely bedridden

None

Forgetting that he/she had eaten and wanted to eat again

Do not know how to choose food

**……**

Delusion


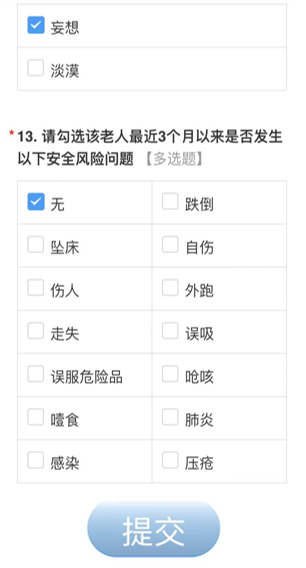


Apathy

**13. Does the dementia people have the following safety risks in the last 3 months?**

None

Falls

Hurting others

Falling out of bed

Sneaking out

Self-injury

Getting lost

Accidental aspiration

Accidental ingestion

Irritating cough

Pressure injury

Infection

Choking on food

Pneumonia

**Submit**

**Figure S2.** The operation flow interface of the “comprehensive evaluation” module.
