# Supplementary material for: Usability Evaluation of a Knowledge Graph–Based Dementia Care Intelligent Recommender System: Mixed Methods Study
Source: J Med Internet Res. 2023 Sep 26;25:e45788. doi: 10.2196/45788 (PMC10565620; doi:10.2196/45788)
Supplement: Multimedia Appendix 3 [file jmir_v25i1e45788_app3.docx]

**Multimedia Appendix 3**


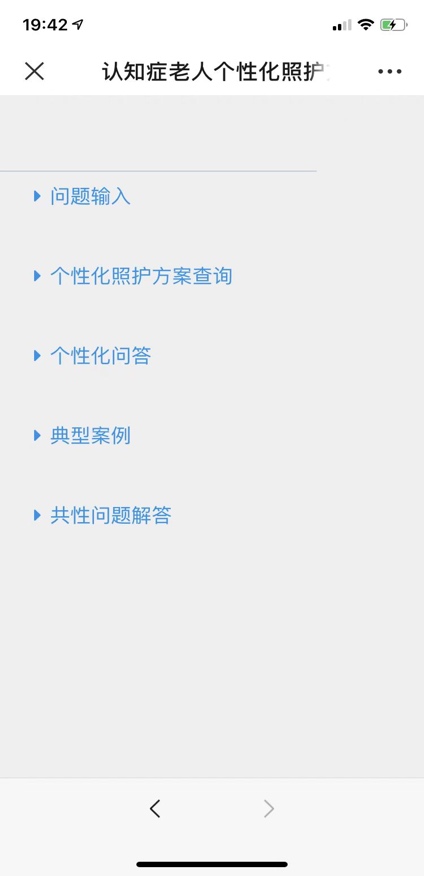


**Dementia care intelligent recommender system**

**Comprehensive evaluation**

**Personalized care plan query**

**Personalized question-answering**

**Typical cases**

**Common questions and answers**


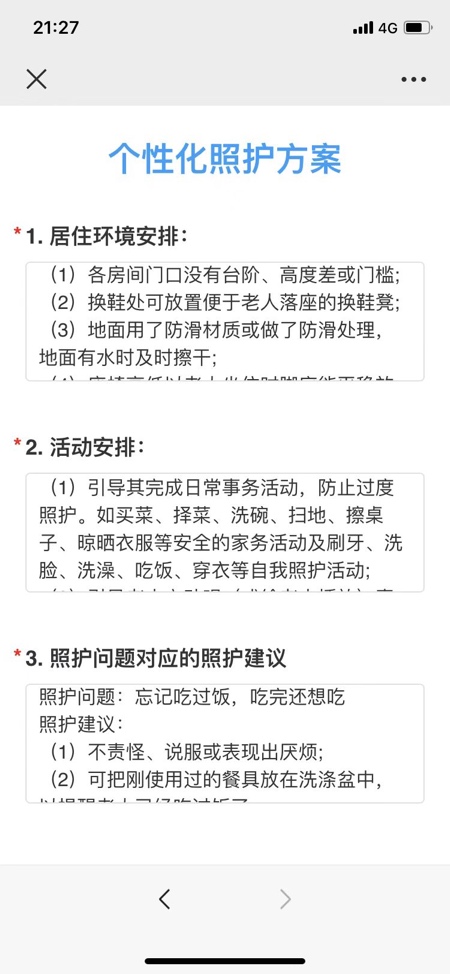


**Personalized care plan**

**Arrangement of the living environment**

**Arrangement of activities**

**Corresponding care advice for specific care problems**

Care advice

Care problems

**Figure S3.** The operation flow interface of the “personalized care plan query” module.
