# Supplementary material for: Usability Evaluation of a Knowledge Graph–Based Dementia Care Intelligent Recommender System: Mixed Methods Study
Source: J Med Internet Res. 2023 Sep 26;25:e45788. doi: 10.2196/45788 (PMC10565620; doi:10.2196/45788)
Supplement: Multimedia Appendix 4 [file jmir_v25i1e45788_app4.docx]

**Multimedia Appendix 4**

**Home page**

**Backstage management**

**center**


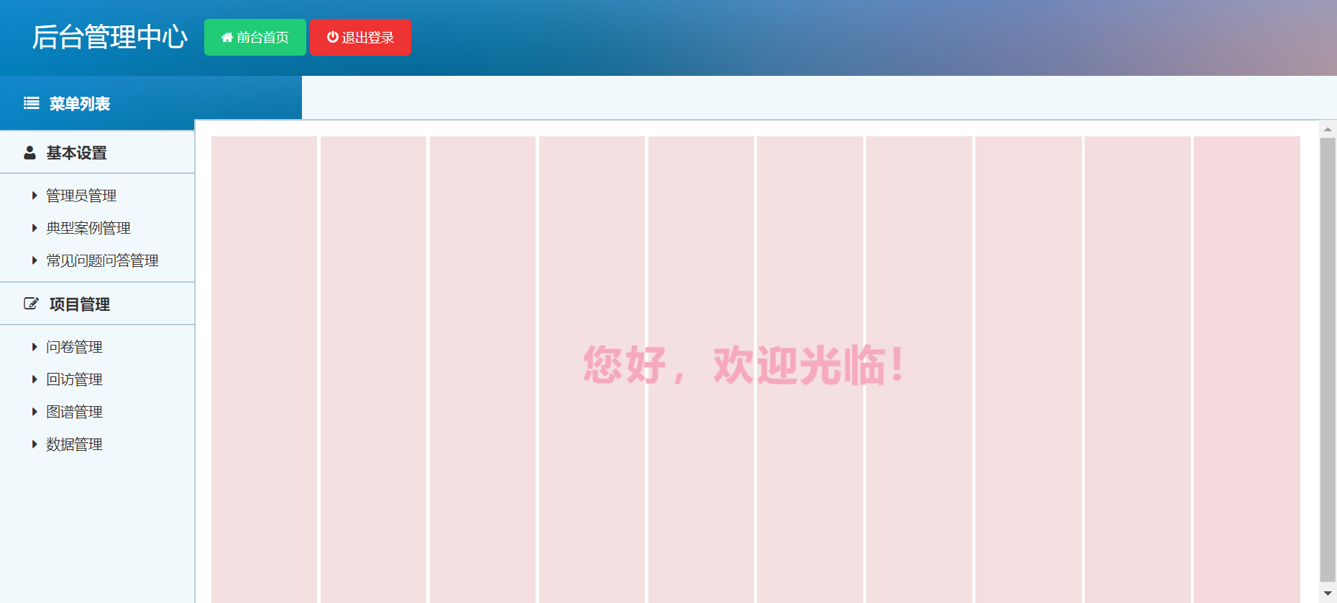


**Menu list**

**Log out**

**Basic setting**

Typical cases management

Administrators management

**Project**

**management**

Common questions and answers management

**Hello, welcome!**

Return visits management

Questionnaires management

Data management

Knowledge graph management

**Figure S4.** The interface of backstage management.
