# Supplementary material for: Usability Evaluation of a Knowledge Graph–Based Dementia Care Intelligent Recommender System: Mixed Methods Study
Source: J Med Internet Res. 2023 Sep 26;25:e45788. doi: 10.2196/45788 (PMC10565620; doi:10.2196/45788)
Supplement: Multimedia Appendix 5 [file jmir_v25i1e45788_app5.docx]

**Table S1.** Results of the interview content analysis

| **Theme** | **Subtheme** | **Authors’ summary** | **Participants’ Quotes** |
| --- | --- | --- | --- |
| **Overall Experience Using the**  **DCIRS** | / | Caregivers generally stated that the content of the DCIRS was professional and comprehensive, the DCIRS was easy to use, and the information was convenient to obtain, which was very helpful to them. | “The overall feeling is fairly good. It can be used on both mobile phones and computers, which is very convenient. I use it on my mobile phone, and the interface is clear and simple after logging into the system.” (C1) |
| **DCIRS Advantages and Beneficial Experiences** | Meeting personalized needs | The performance of each person with dementia was different, and the needs of caregivers also varied. In this interview, 12 caregivers mentioned that the content of the DCIRS was professional and instructive and could meet their personalized needs. | “I think the program is very good. It starts with assessment, and then the care plan given according to the assessment situation is really detailed, personalized, and useful. For example, if people have mild dementia, the plan is for mild dementia; if people have moderate dementia, the plan is for moderate dementia.” (C14) |
|  | Module functions complement each other | Twelve caregivers mentioned that the content of the DCIRS was comprehensive and that the function settings of each module were reasonable, complementary, logical and hierarchical. | “First, there is an evaluation of the overall situation, and then there is a complete care plan. If you have any individual questions, you can also ask them in the ‘personalized question-answering’ module. In addition, typical cases can be used as a supplement. The functional modules are relatively comprehensive. Basically, anything I can think of can be found on this platform.” (C1) |
|  | High authority, credibility, and ease of understanding | Some caregivers reported that the knowledge provided by the DCIRS came from reliable sources and had high authority and credibility and that the content was easy to understand. | “We trust the authority and scientificity of the information on the platform because if we search directly on the internet, we are not sure whether the information is correct.” (C4) |
|  | Convenient, smooth, and easy to use | Some caregivers said it was convenient to use the DCIRS to acquire knowledge, and the operation was smooth and easy. | “This system is all about the disease of dementia. It is more convenient to find the information you want.” (C15) |
|  | Enhancing confidence and decision-making abilities | Some caregivers expressed that the use of the DCIRS could enhance confidence in caring for people with dementia, reduce the sense of uncertainty in the process of care, and improve decision-making abilities. | “I feel an improvement in my confidence in caring. This platform gave me a lot of personalized suggestions, which made me more confident.” (C13) |
|  | Better social understanding and support | Some caregivers said that by using the DCIRS and contacting the research team, they felt that society was gradually paying attention to dementia, and they felt better understood and supported by society. | “Through this platform, I was happy to find that there are so many people paying attention to us. I think society is gradually accepting this disease and understanding our family members and patients, and we won’t always be discriminated against.” (C16) |
| **Shortcomings and** **Optimization Suggestions** | Aesthetic enhancement | Aesthetics played an important role in the online products. Some caregivers thought that the DCIRS interface appearance was not visually warm enough and needed to be further improved. Login via website links was not convenient, and it was suggested that it be moved into WeChat small programs or APPs. | “After logging in, I felt that the interface was not warm enough. The platform should be more beautiful in the visual aspect and look more comfortable. I feel the design is relatively simple.” (C1)  “Every time you enter, you have to click the website link again, unlike in an APP or WeChat small program, which may be more convenient to enter.” (C13) |
|  | Module function optimization | Some caregivers believed that the functions of some modules could be further optimized such as classifying “typical cases” and “common questions and answers” according to disease stages to facilitate finding of suitable content faster. | “In the ‘common questions and answers’ module, some of information was about dementia prevention, and some was about mild dementia. If my patient has moderate dementia, I’ll go through a lot of pages. If these questions belong to the mild, moderate, or severe stages, you can classify them, and then I will click the moderate category to find what I want quickly.” (C13) |
|  | Dynamic evaluation and continuous tracking | Some caregivers proposed that users could be dynamically evaluated and continuously tracked in the future to facilitate the comparison before and after the intervention. In addition, users could be called upon to give feedback on the effectiveness of the care plans pushed by the platform to help optimize the platform. | “After the implementation of these care plans for a period of time, there should be feedback; that is, in the process of implementation, I can think about what is most useful on the platform. For example, 7 out of 10 users say that a care suggestion doesn’t really work, and you might consider not emphasizing it in the future. There are several suggestions that everyone says really work, and you could probably put these at the top of the list.” (C14) |
|  | Enabling interaction | Some caregivers thought it would be helpful if the DCIRS could allow them to interact with professionals or other caregivers. | “Is it possible to add a function so that we can directly communicate with experts, such as asking questions that experts can answer for us? Or, if I have any questions, I could input them in a voice recording, and have the expert respond in the same way. However, this would someone to be there all the time, which could be challenging.” (C11) |
|  | Caregiver support | Some caregivers believed that caring for relatives with dementia is a long process and that caregivers will experience long-term psychological suffering. This platform is mainly used by caregivers, so it is necessary to set up a caregiver support module. | “How should family members of patients make psychological adjustments? Many times, we, as family caregivers, are under enormous psychological pressure and don’t know how to face it. Because this road is so long, it might be good to add a dedicated caregiver support module.” (C8) |
|  | Resource recommendations | Some caregivers needed certain resources and hoped that the platform could recommend specialized dementia care institutions or home service staff, toys or daily supplies suitable for people with dementia, professional psychological counselling, etc. | “For example, in Tianjin, which institutions can accept older people with dementia and who can provide them with home services? Such resource recommendations are expected. We want to find these resources, but there is no way. With these features, we, as users, would prefer to use this system.” (C15) |
| **Willingness to Continue Using the DCIRS** | / | All caregivers expressed their willingness to continue using the DCIRS, and some were willing to continue using the DCIRS because they thought the content was professional, practical and targeted. Some caregivers were willing to continue using it because they thought that the patients would have new problems in the future, and the content on the DCIRS would be updated. However, two caregivers believed that due to their own knowledge reserve or other resources, the subsequent dependence on the DCIRS might not be high. | “I am willing to continue to use it because my own professional knowledge is limited. I can learn a lot of professional knowledge on this platform, which is very targeted. It is specific to our disease and involves almost all aspects.” (C6)  “I have been taking care of patients for many years, and I usually pay much attention to the knowledge of dementia care, which comes from a wide range of sources. I have learned a lot about this disease in the past few years. To be honest, I may not rely on your platform very much in the future.” (C9) |
